# Carbon Eliminated by Plants

**Published:** 1844-11-16

**Authors:** 


CARBON ELIMINATED BY PLANTS.

Dr. Aldridge, from certain experiments which he has performed, draws the conclusion that plants decompose carbonic acid by night as well as by day; in darkness as well as in the light; that the separation of carbon from oxygen, the retention of the former and the disengagement of the latter, is a function performed by all plants under all circumstances, although it is performed with greatly increased energy when vegetables are exposed to the stimulating influence of light.—*Ibid.*

